# Roles of collagen cross-links and osteon collagen/lamellar morphotypes in equine third metacarpals in tension and compression tests

**DOI:** 10.1242/jeb.247758

**Published:** 2024-07-24

**Authors:** John G. Skedros, Michael R. Dayton, John T. Cronin, Chad S. Mears, Roy D. Bloebaum, Xiaodu Wang, Kent N. Bachus

**Affiliations:** ^1^ University of Utah, Department of Orthopaedics, Salt Lake City, UT 84108, USA; ^2^ University of Colorado, Department of Orthopedics, Aurora, CO 80045, USA; ^3^ Department of Mechanical Engineering, University of Texas, San Antonio, TX 78249, USA; ^4^ Research Service, Veterans Affair Medical Center, Salt Lake City, UT 84148, USA

**Keywords:** Secondary osteons, Haversian systems, Collagen fiber orientation, Cortical bone, Bone adaptation, Bone mechanical properties, Energy absorption, Strain-mode-specific testing

## Abstract

Many bones experience bending, placing one side in net compression and the other in net tension. Because bone mechanical properties are relatively reduced in tension compared with compression, adaptations are needed to reduce fracture risk. Several toughening mechanisms exist in bone, yet little is known of the influences of secondary osteon collagen/lamellar ‘morphotypes’ and potential interplay with intermolecular collagen cross-links (CCLs) in prevalent/predominant tension- and compression-loaded regions. Paired third metacarpals (MC3s) from 10 adult horses were prepared for mechanical testing. From one MC3/pair, 5 mm cubes were tested in compression at several mid-shaft locations. From contralateral bones, dumbbell-shaped specimens were tested in tension. Hence, habitual/natural tension- and compression-loaded regions were tested in both modes. Data included: elastic modulus, yield and ultimate strength, and energy absorption (toughness). Fragments of tested specimens were examined for predominant collagen fiber orientation (CFO; representing osteonal and non-osteonal bone), osteon morphotype score (MTS, representing osteonal CFO), mineralization, porosity and other histological characteristics. As a consequence of insufficient material from tension-tested specimens, CCLs were only examined in compression-tested specimens (HP, hydroxylysylpyridinoline; LP, lysylpyridinoline; PE, pentosidine). Among CCLs, only LP and HP/LP correlated significantly with mechanical parameters: LP with energy absorption, HP/LP with elastic modulus (both *r*=0.4). HP/LP showed a trend with energy absorption (*r*=−0.3, *P*=0.08). HP/LP more strongly correlated with osteon density and mineralization than CFO or MTS. Predominant CFO more strongly correlated with energy absorption than MTS in both testing modes. In general, CFO was found to be relatively prominent in affecting regional toughness in these equine MC3s in compression and tension.

## Teaser

**Summary:** Collagen intermolecular cross-links adapt regions in equine third metacarpals for differences in load histories from natural bending. These cross-links help enhance bone toughness, which helps resist fracture.

## INTRODUCTION

**Fig. 1.**
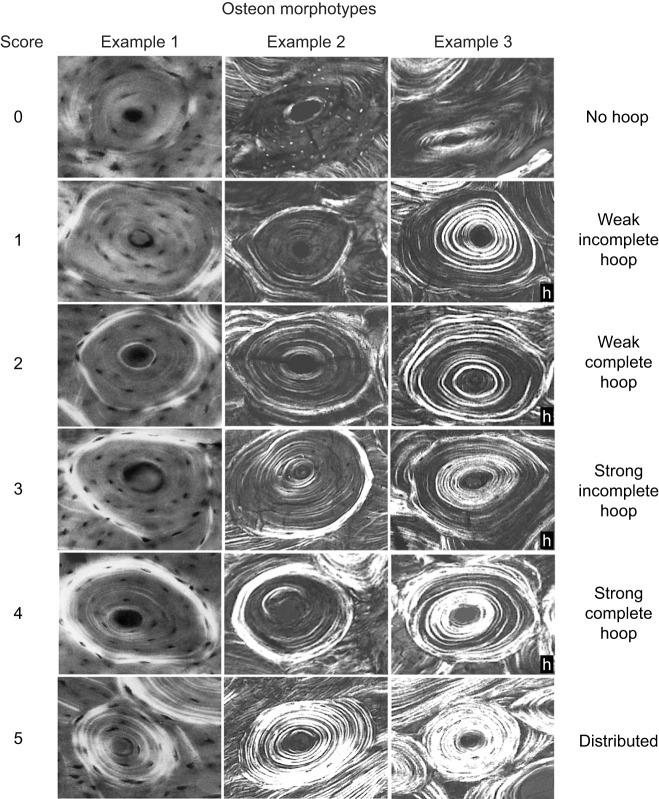
**Six-point osteon morphotype scoring system.** The six-point scoring scheme with examples of each osteon birefringence pattern (osteon collagen/lamellar morphotype) in circularly polarized light. The numerical values of the six morphotypes are used to calculate the osteon morphotype score (MTS) of entire microscopic images that contain many osteons (Skedros et al., 2009, 2011b). Four of the numerical scores shown include consideration of completeness and birefringence strength (brightness) of the peripheral ring ‘O’ or hoop (which is shown most clearly in osteon 4 of example 1 and has relevance in the debonding process that occurs when bone fails; Skedros et al., 2013a): 0=category N, a dark osteon with no birefringent lamellae; 1=category OWI, a combination of OI and OW; 2=category OW, similar to O but the birefringent ring is weak (W); 3=category OI, similar to O but the birefringent peripheral ring is incomplete (I); 4=category O osteon with dark interior and strongly birefringent peripheral lamellae; 5=‘distributed’ osteon group. This group includes ‘bright’ and ‘alternating’ osteons. Note there can be osteons that are considered to be ‘hybrid’ (h) morphotypes because they have variable gray-level patterns within the osteon wall (see the middle four osteon images in example 3 column). These osteons are also scored with the six-number scheme. Images in example 1 column are reproduced from Martin et al. (1996) with permission of Elsevier Science, Inc.

Bones resist fracture by optimizing bone quality (i.e. tissue mechanical properties), quantity and the distribution of bone tissue (Jepsen, 2011; McNerny et al., 2015; Willett et al., 2022). More specifically, a bone's mechanical requirements are ensured by having adequate stiffness, strength, fatigue resistance and fracture toughness (Currey, 2002; Launey et al., 2010; Rosa et al., 2022). Importantly, the critical structural property of bone is its resistance to fracture (Ritchie et al., 2008; Ritchie, 2011). Microdamage accommodation and repair processes in bone, typically via osteonal remodeling (i.e. secondary osteon formation), and load damping/modifying functions of muscles are essential in optimizing a bone's capacity for energy absorption, which is of paramount importance for resisting fracture (Ritchie et al., 2008; Burr, 2011; Zimmermann et al., 2015). Secondary osteons (Haversian systems) are microscopic quasi-cylindrical entities that are formed by the actions of osteoclasts and osteoblasts, and have important biomechanical and metabolic functions (Martin et al., 1998; Skedros et al., 2013b; Chang and Liu, 2022). In this study, we consider how some types of secondary osteons, intermolecular collagen cross-links (CCLs), and other material (i.e. histocompositional) characteristics influence the mechanical properties of cortical (compact) bone in various regions in the mid-shaft area of equine third metacarpals (MC3s). All osteons referred to in this study are secondary osteons, which distinguishes them from primary osteons of unremodeled bone (where primary bone between and in the vicinity of secondary osteons is also known as interstitial bone). Herein, we also refer to osteon collagen/lamellar ‘morphotypes’ that are best detected in circularly polarized light of thin sections (Skedros et al., 2013a) and can be distinguished from other osteon types that have distinctive morphological characteristics that do not include collagen/lamellar variations (Skedros et al., 2007) (Fig. 1). Healthy bone can mitigate initiation and propagation of microcracks because of interfaces within and at the periphery of osteons (i.e. cement lines) while enabling the osteonal remodeling processes to repair the affected bone volume. The mineralization of individual osteons, the accumulation of collagen cross-links within the typically more mineralized interstitial bone, and the stiffness mismatch at cement lines all contribute to tissue heterogeneity that provides beneficial toughening mechanisms of bone (Stockhausen et al., 2021).

A main goal of this study was to examine cortical (compact) bone of adult equine MC3s for what are believed (1) or known (2) to be two important toughening mechanisms, respectively: (1) regional variations in the distribution of secondary osteon collagen/lamellar ‘morphotypes’ and (2) intermolecular CCL densities. Little is known about the role of osteon morphotypes and their potential synergistic interactions with CCLs in this context. To our knowledge these two material characteristics have not been studied in the context of how they might help accommodate regional variations in the tension versus compression strain modes that occur habitually in opposing cortices of many bones as the result of prevalent/predominant, often directionally consistent, bending loads. A bone's capacity to adapt its material organization for the regional differences in net tension versus net compression can be revealed by strain-mode-specific (SMS) testing; namely, compression testing of bone from regions habitually loaded in compression and tension testing of bone from regions habitually loaded in tension. A review of studies that have used SMS testing to reveal bone material/tissue adaptations to the non-uniform strain-mode distribution engendered by habitual bending can be found in Skedros et al. (2024). We used regional SMS mechanical testing in this study.

**List of abbreviations**

CCL: collagen cross-link
CFO: collagen fiber orientation
FASB: fractional area of secondary bone
HP: hydroxylysylpyridinoline
LP: lysylpyridinoline
MC3: third metacarpal
MTS: morphotype score
OA: osteon area
OLPD: osteon lacuna population density
OPD: osteon population density
PE: pentosidine
SMS: strain mode specific
WMGL: weighted mean gray level

Regional variations in osteon morphotypes have been shown to correspond to non-uniform strain-mode distributions (e.g. net tension versus compression in mutually exclusive cortical locations) that are linked to generally directionally consistent bending that many limb bones experience (Skedros et al., 2009, 2011a, 2013a; Skedros, 2024). For example, they have been described in human and chimpanzee proximal femoral diaphyses (subtrochanteric region) where they are thought to help accommodate a high bending moment that produces net compression in the medial cortex (where ‘compression-adapted morphotypes’ are prevalent) and net tension in the lateral cortex (where ‘tension-adapted morphotypes’ are prevalent) (Skedros et al., 2011b, 2013a). It has been speculated that these regional distributions of specific osteon morphotypes help curb potentially deleterious differences in the incidence of microdamage that can occur between opposite sides of a bone as a result of habitual differences in prevailing strain mode (Reilly and Currey, 1999; Skedros et al., 2011b). This reflects the fact that although there are mechanical and physiological benefits of habitual bending (Biewener et al., 1983; Bertram and Biewener, 1988; Skedros, 2024), loading bone in tension can be relatively deleterious when compared with compression (Reilly and Currey, 1999; Burr, 2011; Skedros, 2024). In healthy adult humans and other mammals, it is speculated that osteon morphotypes help to avoid more common stress fractures by differentially toughening regions of the same bone for tension strains in some locations and compression strains in others (Nunamaker, 2001; Skedros et al., 2013a, 2014). For example, Nunamaker (2001) suggested that the failure to achieve this regional microstructural toughening (typically via osteonal remodeling) is what leads to the high incidence of stress fractures in the MC3 of Thoroughbred racehorses. However, little is known regarding the effects that differences in the population densities of specific osteon morphotypes have on the mechanical properties of cortical bone. The only studies that have explored these issues have focused primarily on the failure-related debonding behavior of some of these osteon morphotypes (Hiller et al., 2003; Bigley et al., 2006).

Independent of osteons, the integrity of the collagen network in bone tissue is also integral for adequate toughness of bones and has been implicated in age-related decreases in toughness (Wang et al., 2002; Burr, 2011; Zimmermann et al., 2015; Willett et al., 2022). Collagen cross-links, which are intermolecular bonds in the collagen component of bone, can influence the capacity of bone to sustain plastic deformation before breaking (Gauthier et al., 2018). For example, it has been shown that decreases in collagen cross-link concentration are associated with decreases in bone stiffness and the capacity to absorb energy to fracture (Wang et al., 2001). Intermolecular covalent-bond CCLs are important in this context and include the enzymatic cross-links hydroxylysylpyridinoline (HP) and lysylpyridinoline (LP) and non-enzymatic CCLs often measured by pentosidine (PE) concentration (Wang et al., 2002; Saito et al., 2006; Nyman et al., 2007; McNerny et al., 2015; Willett et al., 2022). Investigation of CCLs and the potential mechanical interplay with other histocompositional characteristics in the context of specific mechanical properties of bone may help clarify their relative contributions in accommodating the non-uniform loading environment that limb bones typically experience. For example, studies conducted using limb bones from horses and deer have shown that regional variations in predominant collagen fiber orientation (CFO) strongly influence energy absorption of cortical bone in the physiological context of SMS testing (Skedros et al., 2006, 2024). Although studies of various bones from many mammalian species have shown that predominant CFO correlates strongly with osteon morphotype score (MTS) (Fig. 1) (reviewed in Skedros, 2024), the mechanical influences of regional variations in osteon MTSs have not been studied. In the present study, we examined the relative influences of three CCLs, variations in osteon MTSs and variations in other often-studied histocompositional characteristics on the mechanical properties of cortical bone in SMS and non-SMS compression testing of mid-diaphyseal bone of the equine MC3, which has served as a model for understanding how bone tissue adapts to avoid stress fractures associated with a harsh loading environment with habitual directional bending (Biewener et al., 1983; Gibson et al., 1995; Martin et al., 1996, 1997; Nunamaker, 2001; Skedros et al., 2006).

**Fig. 2.**
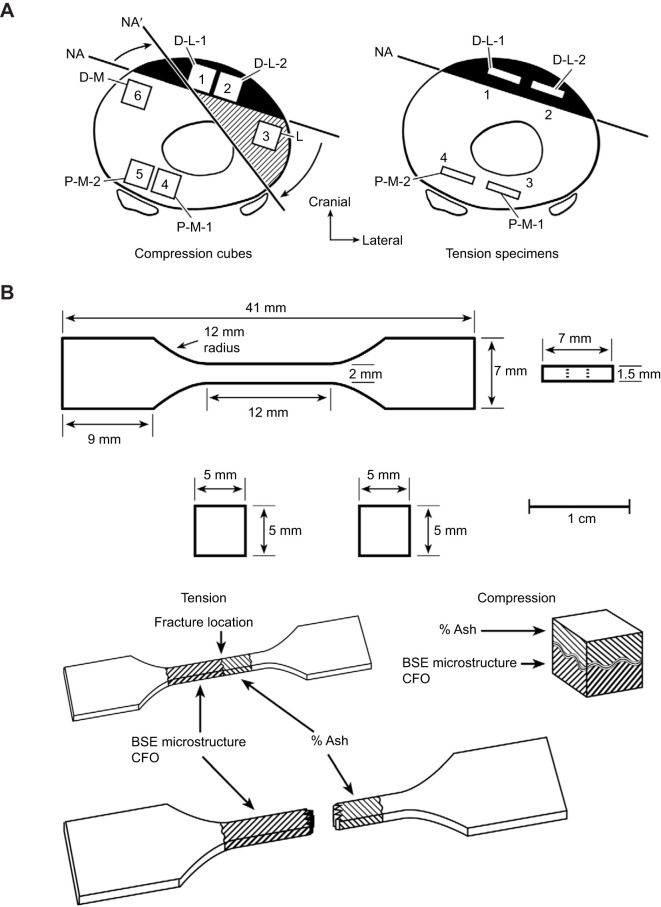
**Transverse sections of a third metacarpal (MC3) and test specimens.** (A) Drawings of transverse sections showing the locations of an MC3 where compression (left) and tension (right) test specimens were obtained: D, dorsal; L, lateral; M, medial; and P, palmar. NA, neutral axis. Black regions show where tension is prevalent/predominant; white regions indicate areas of habitual compression. The curved arrows in the top left drawing show rotation of the neural axis that occurs at faster gait speeds, which causes the lateral cortex (hatched region) to change from its ambient compression strain mode to tension (i.e. a strain-mode reversal occurs). (B) The dimensions of the tension (dumbbell-shaped) and compression (cubic) specimens. The hatched areas show regions of the specimens where histocompositional analyses were performed. BSE, backscattered electron imaging. Figure reproduced with permission from Skedros et al. (2006).

We used the same 10 adult equine MC3s from our prior studies where we examined the concept of ‘regional safety factors’ (Skedros et al., 2003) and the influences of various bone material characteristics on the regional mechanical properties of machined specimens obtained from several locations of the mid-diaphysis of each bone that were each independently tested in tension and compression (i.e. SMS and non-SMS tests) (Fig. 2, top) (Skedros et al., 2006). The regional material characteristics examined in our prior study (Skedros et al., 2006) included: secondary osteon population density, fractional area of secondary bone, cross-sectional size of individual secondary osteons, porosity, osteocyte lacuna population density, predominant CFO and mineral content (by ashing). The mechanical parameters examined in these two prior studies included: Young's elastic modulus, yield stress, ultimate stress and energy absorption (toughness; including pre-yield, post-yield and total, as defined by areas under the stress–strain curve) (Turner and Burr, 1993). In the present study, we used fragments of the mechanically tested cube (compression) and dumbbell (tension) bone specimens (Fig. 2, bottom) from these two prior studies as the foundation for examining the mechanical influences of CCL density and osteon MTSs. The material characteristics of the fragments from these mechanically tested specimens were examined with a specific focus on energy absorption (toughness) data because this is the mechanical parameter most likely influenced by osteon morphotypes and CCLs (Wang et al., 2001; Saito et al., 2006; Skedros et al., 2006; Depalle et al., 2015) and is more important in the assessment of fracture risk than bone stiffness and strength (Burr, 2011; Zimmermann et al., 2015). Consequently, the same fragments of the tension- and compression-tested specimens that we previously examined for predominant CFO and the other microstructural and material characteristics mentioned above were re-examined statistically in the present study with the inclusion of osteon MTS and CCL data from all of the regions shown in Fig. 2 (Skedros et al., 2006). Using the same microscopic images from which CFO data were previously obtained and reported, we quantified osteon MTS (representing osteonal CFO; Fig. 1) for each of the tension- and compression-tested specimens. Because of insufficient residual material from tension-tested specimens, only compression-tested specimens were examined for CCLs (HP, LP and PE) (Wang et al., 2002; Nyman et al., 2007).

**Fig. 3.**
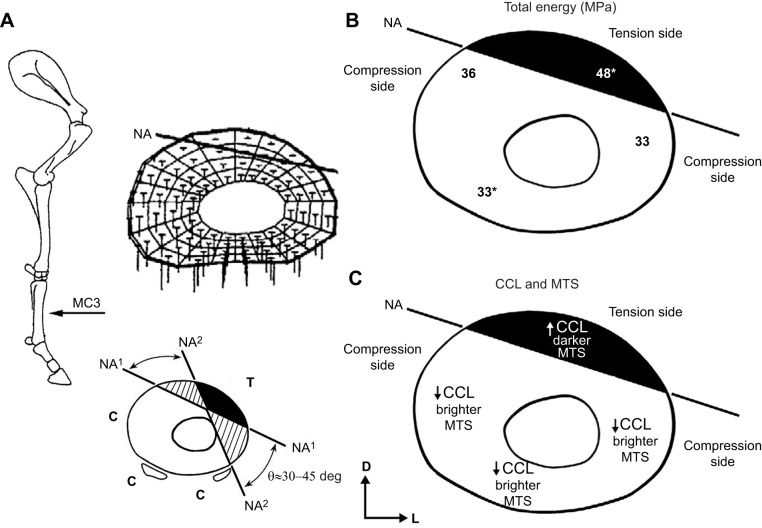
**Forelimb and mid-diaphyseal cross-section of the MC3 of a Thoroughbred.** (A) Top: equine forelimb and mid-diaphyseal cross-section of the MC3 showing regional differences in strain magnitude and mode (i.e. tension and compression) and the neutral axis (NA) at mid-stance in accordance with the strain distribution of the finite element model of Gross et al. (1992) as shown above. D, dorsal; L, lateral (for all four cross-sections shown). Bottom: toward the end of stance phase and especially at higher gait speeds the NA rotates clockwise (NA^1^ to NA^2^), which places the lateral cortex in more prevalent/predominant tension (T) (C, net compression) (Nunamaker, 2001) (figure reproduced with permission from Skedros et al., 2007). (B) Mid-diaphyseal cross-section of the MC3 showing regional differences in total energy absorption data from Skedros et al. (2006). The asterisks indicate two regions that are significantly different from each other (*P*≤0.05). (C) Mid-diaphyseal cross-section of the MC3 demonstrating our hypotheses concerning regional differences in collagen cross-links (CCLs) and osteon MTSs (see Table 1).

Table 1 lists the four main hypotheses and additional predictions of the present study, and prior studies that support their formulation. Anticipated regional variations in CCLs and osteon morphotypes that correspond with these predictions are shown in Fig. 3. The four main hypotheses are as follows. (1) Enzymatic CCLs (HP, LP, HP/LP) enhance compressive toughness, which is supported by experimental studies listed in Table 1. (2) Enzymatic CCLs and CFO will be positively correlated. This is based on observations showing that: (i) CFO generally becomes more oblique-to-transverse with the maturation of osteonal and interstitial bone while CCLs are also simultaneously accumulating, and (ii) indirect evidence supporting the idea that collagen fiber alignment can be influenced by the density and/or type of CCLs (Xu et al., 2011; Stockhausen et al., 2021; Wohlgemuth et al., 2023; Skedros, 2024). (3) Enzymatic CCLs and osteon MTS will be positively correlated, which is similarly based on evidence noted in hypothesis 2. (4) Osteon MTS will out-perform CFO in influencing energy absorption because it is closely related to osteon population density (which includes interfaces that help attenuate microdamage propagation, hence enhancing bone toughness) (Burr, 2011; Nobakhti et al., 2014; Zimmermann et al., 2015).

**Table 1.**
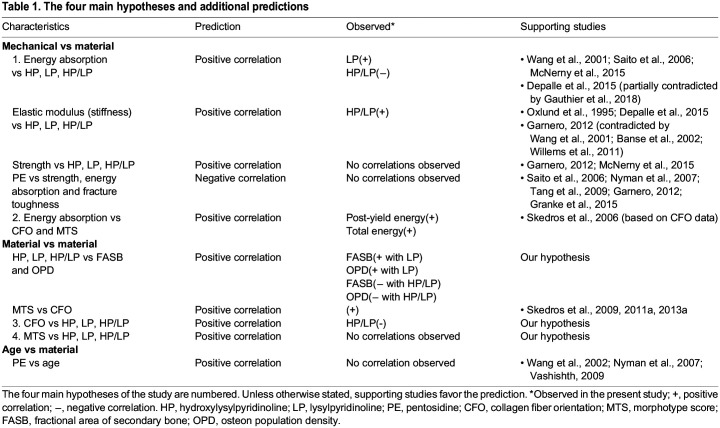
The four main hypotheses and additional predictions

## MATERIALS AND METHODS

### Specimens and mechanical testing

Right and left MC3s from 10 skeletally mature horses, representing a broad age range and with no racing history, were prepared for mechanical testing as described in our previous studies (Skedros et al., 2003, 2006). This study was approved by the University of Utah IACUC (no. 09-06005). The animals were obtained from a regional abattoir where only general information regarding the cause of death of the animals could be obtained but none of the selected animals had gross evidence or history of musculoskeletal disease. Available data regarding animal age, sex and breed are shown in Table 2 (the mass of each animal was not known). Fig. 2 shows the locations from where the specimens for mechanical testing were obtained, and the dimensions of the cubes used in compression tests and the dumbbell-shaped specimens used in tension tests.

**Table 2.**
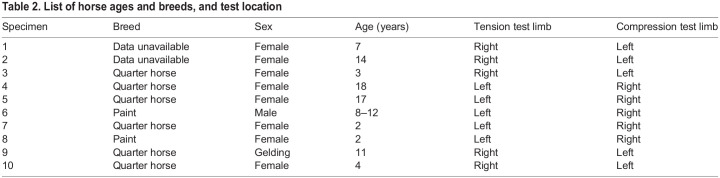
List of horse ages and breeds, and test location

The six 5 mm×5 mm×5 mm cubes for compression testing from one MC3 of each horse were cut from transverse segments obtained in these cortical regions: dorsal–lateral (*n*=2; 20 total specimens), lateral (*n*=1; 10 total specimens), palmar–medial (*n*=2; 20 total specimens) and dorsal–medial (*n*=1; 10 total specimens). From contralateral MC3s, a 50 mm thick segment was sectioned transversely above and below the 50% bone length location. Rectangular slabs were milled to dumbbell-shaped specimens that were 41 mm long and 1.5 mm thick (for tension tests, a detailed drawing of the dumbbell specimens can be seen in Fig. 2). Hence, there were two dumbbells from the dorsal–lateral (19 total specimens) cortex and two dumbbells from the palmar–medial (19 total specimens) cortex. One specimen was discarded from each location because of breakage during milling. This resulted in 60 total specimens for compression testing and 38 total specimens for tension testing. As described below, some specimens were discarded because of aberrant breakage during the testing process.

An Instron Model 4303 (Canton, MA, USA) was used to compress the cubic specimens to failure along the longitudinal diaphyseal axis at a strain-controlled rate of 0.001 s^−1^ (Skedros et al., 2006). Tension data were obtained using an extensometer (MTS^®^ 632.13F-20) attached to each dumbbell-shaped specimen while being loaded to failure at a strain-controlled rate of 0.01 s^−1^ (Skedros et al., 2006). Therefore, habitual/natural tension- and compression-loaded regions were independently tested in compression and tension.

As also described previously, fragments from the fractured specimens (any portion of the cubes or the ‘neck’ portions of the dumbbells) were analyzed for various microstructural characteristics and mineral content (% ash). During tensile testing, premature catastrophic failure and/or failure outside the region between the extensometer clips resulted in seven specimen exclusions from the dorsal–lateral region and two specimen exclusions from the palmar–medial cortex. This reduced the sample size from 19 to 12 in the dorsal–lateral cortex and from 19 to 17 in the palmar–medial cortex. Three compression specimens from the lateral cortex were also excluded for premature catastrophic failure, reducing the sample size from 10 to seven in this specific region. Only nine specimens had tension test data and sufficient residual material to obtain osteon MTSs, which is why the SMS tension sample for the mechanical versus material comparisons is limited to nine specimens. Sample sizes for specific paired comparisons are reported below.

### Analysis of material characteristics

Predominant CFO for each specimen is expressed as a weighted mean gray level (WMGL) in circularly polarized light images of thin ultra-milled sections obtained from each tested specimen (with sectioning in the transverse plane with respect to the long axis of the bone/specimen) (Skedros et al., 2009). For each image, osteon MTSs were also determined using the six-point method (Skedros et al., 2011a) (Fig. 1). As noted, the data for the additional material characteristics from each of the tested bone specimens that we also consider herein were obtained previously (Skedros et al., 2006) and include: osteon population density (OPD), fractional area of secondary bone (FASB), osteocyte lacuna density, osteon area (OA, of individual osteons) and mineralization by ashing (% ash).

Quantitative analysis of CCL density was performed as originally described by Bank et al. (1997) and reported by Wang et al. (2002) in bone specimens. In brief, fragments of compression bone specimens (10 mg) were hydrolyzed at 110°C in 500 µl 6 mol l^−1^ HCl for 20 h and then vacuum dried. Again, owing to insufficient material from tension-tested specimens, only compression-tested specimens were examined for CCLs. The hydrolyzate was then dissolved in water (50 µl mg^−1^ bone) containing 10 nmol pyridoxine ml^−1^ and 2.4 µmol homoarginine ml^−1^ as internal standards and diluted 5-fold in diluent [0.5% (v/v) heptafluorobutyric acid in 10% (v/v) acetonitrile]. Two-step isocratic chromatography was then performed using an HPLC (Waters 600S controller, 626 pump, running Millenium^32^ software) and programmed fluorimeter (Waters 474 scanning fluorescence detector) as described by Bank et al. (1997). The column used was a Waters S5 ODS2 column (4.6 mm×150 mm), packed with 5 µm spherical silica particles with 80 Å pores. The concentration of hydroxylysylpyridinoline (HP), lysylpyridinoline (LP) and pentosidine (PE) cross-links was calculated from the chromatograms and the data for each converted to pmol mg^−1^ bone. These values were then normalized to the collagen content (see below) of the sample.

A portion of the acid hydrolyzate, prepared as described above, was neutralized with sodium hydroxide and then analyzed for its hydroxyproline content according to the colorimetric method described by Woessner (1961). The hydroxyproline content of each sample was determined from a standard curve prepared using l-hydroxyproline. The amount of collagen per mg bone was calculated from the content of hydroxyproline per mg bone by multiplying by a factor of 7.14, as this amino acid represents approximately 14% of the total amino acid pool found in collagen.

### Data segregations (SMS and non-SMS tests) and statistical analyses

The equine MC3 typically experiences net compression in the palmar–medial and dorsal–medial cortices and net tension in the dorsal–lateral cortex. The total sample size of 98 specimens (60 compression, 38 tension) was segregated into subsets of data based on this known habitual loading environment of the equine MC3 and the testing mode (i.e. SMS versus non-SMS testing) (Skedros et al., 2006, 2024). This resulted in six additional subsets of data that were statistically evaluated separately, in addition to the statistical analysis of the total sample size of 98 specimens. The six additional data subsets are as follows: (1) non-SMS compression including the lateral cortex, (2) non-SMS compression without the lateral cortex, (3) non-SMS tension, (4) SMS compression with the lateral cortex, (5) SMS compression without the lateral cortex and (6) SMS tension. The dorsal–lateral and/or lateral cortical regions of the equine MC3 have been shown to experience net tension or net compression at different gait speeds (Nunamaker, 2001) (Fig. 3A). Hence, this and relatively more longitudinal CFO toward the more lateral aspect of the bone provide the rationale for the additional subsets that excluded the lateral cortex from other ‘compression regions’ (Skedros et al., 1996, 2006, 2007).

**Fig. 4.**
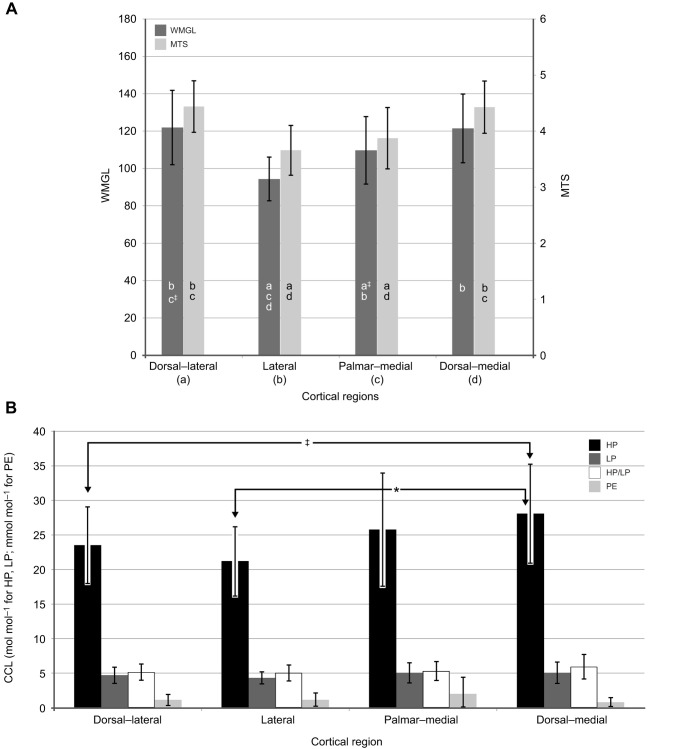
**Regional differences in predominant collagen fiber orientation (CFO), osteon MTS and CCL data.** (A) Predominant CFO (expressed as weighted mean gray levels, WMGLs) and osteon MTS – results of paired comparisons between regions. Data (means±s.d.) were obtained exclusively from compression-tested specimens to allow for easier comparison with regional differences in CCLs. Letters in bars (corresponding to the cortical regions indicated) show significant differences (*P*<0.05) in paired comparisons of each characteristic (‡ indicates a trend). (B) CCL data – results of paired comparisons between compression-tested regions. HP, hydroxylysylpyridinoline; LP, lysylpyridinoline; PE, pentosidine (note the change in units for PE). The sample sizes for the two-sample *t*-tests in A and B are: (1) dorsal–lateral: 16–20 for each parameter, (2) lateral: 7–10 for each, (3) palmar–medial: 9 for CFO and 16–18 each for MTS and CCLs, and (4) dorsal–medial: 8 for MTS, 10 for CCLs, and 18 for CFO. ^‡^0.06≤*P*≤0.09; *P≤0.05.

Pearson correlations were used to detect significant relationships between osteon MTS, predominant CFO (expressed in terms of WMGLs in polarized light images), each of the other material characteristics and each of the six mechanical properties. Two-sample *t*-tests were used to assess regional variations in the new data reported herein (osteon MTSs and CCLs), with an emphasis on examining relationships with the previously reported CFO data in addition to the other mechanical and material parameters from our prior studies (Fig. 4). Percentage difference was calculated as 100×(difference/mean) for data from a paired comparison (Cole and Altman, 2017). Statistical significance was set at *P*≤0.05. Statistical trends are also reported in Tables S1–S4. In accordance with a study of CCLs, porosity, and mineral and collagen content in 17 human tibiae from our affiliated laboratory, we also used a general linear model (GLM) to determine which material characteristics best explained variance in each mechanical parameter (Nyman et al., 2007). This analysis included the appraisal of interactions, and factors with the highest *P*-value were sequentially removed to obtain the highest *R*^2^ value. Unfortunately, the statistical power in the present study is insufficient (because of insufficient sample sizes) to adequately perform this analysis. The statistical procedures used the NCSS statistics package (NCSS 2020, NCSS LLC, Kaysville, UT, USA; NCSS.com).

## RESULTS

The results reported below focus on data from the SMS tests and the hypotheses/predictions (Table 1) related to the main material characteristics of this study [i.e. predominant CFO, osteon MTS and the four CCL parameters (LP, HP, HP/LP and PE)]. Comparisons between material characteristics versus mechanical parameters and material versus material characteristics were examined for significant differences (*P*≤0.05) and trends (0.05<*P*≤0.1) (Table 3). CFO data from our previous study (Skedros et al., 2006) are also specifically reconsidered because of the close relationship with osteon MTS (Skedros et al., 2009). The myriad of other considerations (including analysis of non-SMS tests) can be found in Tables S1–S4. Unless otherwise noted, the data subsets that are primarily considered here are: (1) SMS compression (palmar–medial and dorsal–medial cortices), and (2) SMS tension (dorsal–lateral cortex). In some cases, results dealing with non-SMS data subsets are also reported below. With respect to the main hypotheses and predictions (Table 1), our results showed the following. (1) Among all CCL characteristics, only LP significantly correlated with an energy absorption parameter (positive correlation with total energy) when considering SMS compression-tested specimens. HP/LP showed negative trends with post-yield energy and total energy absorption (both *P*=0.08) (Table 4). (2) Among all CCL characteristics, only HP/LP correlated with CFO (correlation was also negative) when considering all non-SMS compression-tested specimens (including the lateral cortex) (Table S3). (3) No significant correlations were detected for each of the enzymatic CCLs (LP, HP and HP/LP) and osteon MTS (Table 5). (4) When considering all six subsets of mechanical testing data (including tension and compression tests), CFO was more strongly correlated with energy absorption compared with osteon MTS (Table 4; Tables S1 and S2). (5) Osteon MTS and/or CFO positively correlated with one or more energy absorption parameters (pre-yield energy, post-yield energy, total energy) in all mechanical testing data subsets except the non-SMS tension group (Table 4; Tables S1 and S2). (6) None of the CCLs (including PE) significantly correlated with age in any of the mechanical testing subsets (Table S3).

**Table 3.**
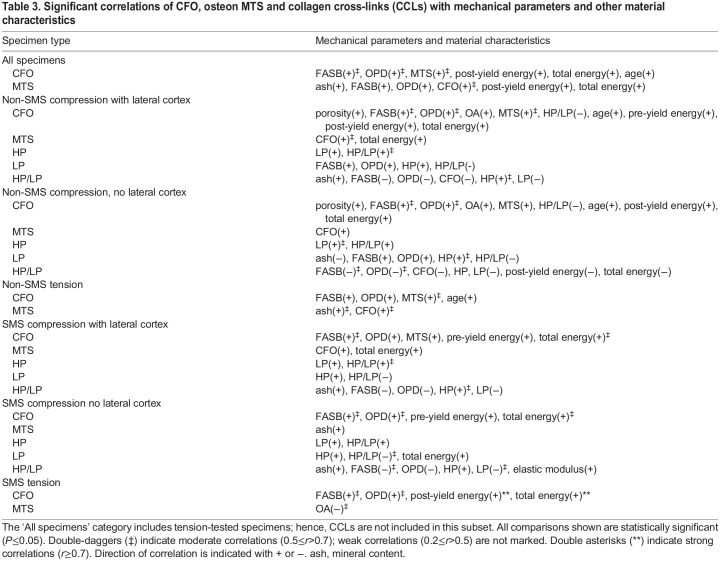
Significant correlations of CFO, osteon MTS and collagen cross-links (CCLs) with mechanical parameters and other material characteristics

**Table 4.**
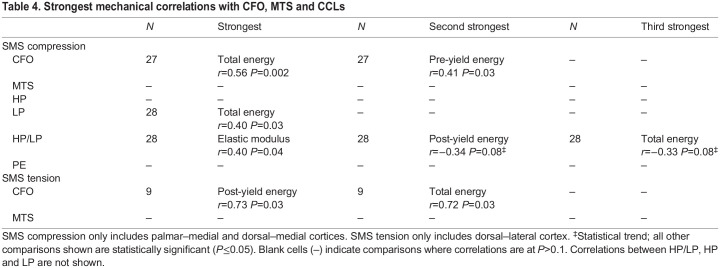
Strongest mechanical correlations with CFO, MTS and CCLs

**Table 5.**
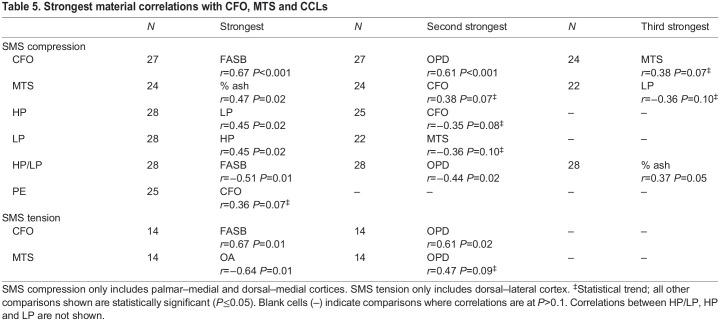
Strongest material correlations with CFO, MTS and CCLs

### Correlation analysis of mechanical parameters versus CFO, osteon MTS and CCLs

In the context of SMS compression tests (palmar–medial and dorsal–medial regions), the top two strongest mechanical correlates with CFO were total (*r*=0.56, *P*=0.002) and pre-yield (*r*=0.41, *P*=0.03) energy absorption (Table 4). No significant correlations with mechanical parameters were found for osteon MTS in either testing mode.

LP significantly correlated with total energy absorption (*r*=0.40, *P*=0.03) but not with any other mechanical parameter. The top three strongest correlates with HP/LP were elastic modulus (*r*=0.40, *P*=0.04), post-yield energy absorption (*r*=−0.34, *P*=0.08) and total energy absorption (*r*=−0.33, *P*=0.08) (Table 4). Notably, the negative statistical trends (0.05<*P*≤0.09) between HP/LP and post-yield and total energy absorption become significant when considered in the context of non-SMS compression testing without the lateral cortex (which is presumably a net ‘tension region’ during peak loading but less clearly than the dorsal–lateral ‘tension region’) (Table S2) (Fig. 3A, bottom) (Skedros et al., 1996, 2006).

For SMS tension tests, the top two correlates with CFO were post-yield energy (*r*=0.73, *P*=0.03) and total energy absorption (*r*=0.72, *P*=0.03), which were the strongest correlations found in this study (Table 4). In contrast, osteon MTS did not correlate with any mechanical parameter in SMS tension tests.

### Correlation analysis of CFO, osteon MTS and CCLs versus other material characteristics

#### CCLs

In the context of SMS compression tests, HP/LP demonstrated the most statistically significant correlations with other non-CCL material characteristics when compared with the other CCL characteristics (HP, LP and PE). The top three correlates for HP/LP were FASB (*r*=−0.51, *P*=0.01), OPD (*r*=−0.44, *P*=0.02) and % ash (*r*=0.37, *P*=0.05) (Table 5). HP was significantly correlated with LP (*r*=0.45, *P*=0.02) and showed a statistical trend with CFO (*r*=−0.35, *P*=0.08). LP showed a statistical trend with osteon MTS (*r*=−0.36, *P*=0.10). PE showed a statistical trend with CFO (*r*=−0.34, *P*=0.09) (Table 5).

#### Osteon MTS and CFO

Relationships between osteon MTS and CFO were explored for statistical significance because these characteristics are most strongly associated. In the context of the SMS compression test subset, the correlation of CFO with MTS was weak (statistical trend: *r*=0.38, *P*=0.07) (Table S3); however, this relationship exhibited the strongest correlation among material characteristic comparisons when all samples (SMS and non-SMS compression and tension tests) were considered (*r*=0.63, *P*<0.01) (Table S1). Also, because osteon MTS is also related to OPD and FASB (i.e. all are osteonal parameters), we focused on comparisons among these characteristics. Examination of data from the SMS compression test subset revealed that: (1) CFO was significantly correlated with FASB and OPD (*r*=0.67 and 0.61, *P*<0.001) and showed a weak trend with osteon MTS (*r*=0.38, *P*=0.07) (Table 5), (2) osteon MTS was significantly correlated with % ash (*r*=0.47, *P*=0.02) and (3) LP showed a statistical trend with osteon MTS (*r*=−0.36, *P*=0.10) (Table 5). In this compression SMS data subset, only CFO showed potential relationships with HP (*r*=−0.35, *P*=0.08), PE (*r*=0.36, *P*=0.07) and HP/LP (*r*=−0.34, *P*=0.09) (Table S3).

When considering non-SMS compression test subsets (with and without the lateral region), LP was significantly positively correlated with FASB and OPD, while HP/LP was significantly negatively correlated with FASB and OPD (Table S3). HP did not show any significant correlation or trend with FASB and OP, which suggests that the density of LP increases as the percentage of remodeled bone increases (i.e. as the population density of secondary osteons increases).

### Regional variations revealed by paired comparisons

As reported previously (Skedros et al., 2006), regional porosity variations were minor in our sample of 10 MC3s; hence, normalization of the mechanical properties data for porosity variations was deemed unnecessary (Zimmermann et al., 2015).

Fig. 4A shows that the lateral cortex (presumably a ‘tension-adapted’ region) MTS was significantly lower than that of the dorsal–medial region, but was not significantly lower than the MTS of the palmar–medial region (note that both dorsal–medial and palmar–medial regions are considered compression adapted). Also, as reported previously, CFO variations were also not strongly SMS in the habitual tension and compression regions shown in Fig. 2 (Skedros et al., 2006). For example, the percentage difference between dorsal–lateral and dorsal–medial regions was <0.5% for CFO and MTS. Additionally, in that regional comparison, only two of the 10 MC3s had a CFO difference that exceeded 20% where statistically significant changes in mechanical properties are likely (Skedros et al., 2006). In contrast, Fig. 4A shows statistically significant percentage differences between lateral and dorsal–medial regions for CFO that exceed 20% in seven of the 10 bones (mean difference 25%), and osteon MTS showed a 17% mean difference. Therefore, regional CFO differences are mechanically significant, and appear to be SMS, in the lateral versus dorsal–medial comparison but not in the dorsal–lateral versus dorsal–medial comparison.

Fig. 4B shows that the concentration of HP tended to be lower in the dorsal–lateral and lateral regions. The regional difference of HP concentration was 18% for the dorsal–lateral versus dorsal–medial comparison (a statistical trend) and 28% for the lateral versus dorsal–medial comparison (*P*<0.05). In contrast, LP, HP/LP and PE showed only very minor regional variations.

## DISCUSSION

We pursued the present study to test the hypothesis that different osteon collagen/lamellar morphotypes (expressed as osteon MTSs) and/or intermolecular CCLs are important correlates of specific mechanical properties in adult equine MC3s. Specifically, we predicted that regional variation in these material characteristics would help optimize energy absorption for the local, regionally prevalent, strain mode (tension versus compression) as seen in many bones subjected to habitual bending (Reilly and Currey, 1999; Hiller et al., 2003; Bigley et al., 2006; Skedros et al., 2013a, 2024). But none of those prior studies of equine MC3s examined these bone material characteristics in machined specimens that are often used in conventional tests that quantify basic/standard mechanical parameters such as stiffness (elastic modulus), strength and energy absorption. Our results add to a growing body of experimental data that reveal an important role for predominant CFO, collagen content and/or intermolecular CCLs in affecting energy absorption in bone (Wang et al., 1998; Banse et al., 2002; Burr, 2002; Nyman et al., 2007; Burr, 2011).

An important finding of our study is that the ratio of the enzymatic CCLs (HP/LP) was significantly correlated with elastic modulus and showed a statistical trend with post-yield and total energy absorption. While HP/LP was the strongest correlate with post-yield energy absorption, % ash was not one of the top two correlates (Table S4). This contrasts with the results from our prior study where % ash was the strongest correlate (and statistically significant) with post-yield energy in SMS compression testing (Skedros et al., 2006). HP/LP was the second strongest (and statistically significant) correlate in elastic modulus in SMS compression testing (% ash was the strongest correlate in this context) (Table S4). Perhaps the positive relationship of HP/LP with elastic modulus is more strongly related to the significant correlation of HP/LP with % ash – where % ash is a well-known strong correlate of tissue stiffness in many bones of many species (Currey, 2002).

Additionally, when considering SMS compression tests, total energy absorption significantly positively correlated with LP (*r*=0.40, *P*=0.03) and, as noted, showed a negative trend with HP/LP (*r*=−0.33, *P*=0.08) (Table 4). HP showed no significant or trend relationship with total energy absorption. This suggests that LP, when compared with HP, is relatively more important in the context of total energy absorption. In other words, as LP increases (and HP/LP deceases as a result of LP being in the denominator), total energy absorption increases as well. However, neither HP/LP nor LP is in the top two correlates in this context (LP is third strongest for total energy absorption and HP/LP is fourth strongest) (Table S2). The statistical trends in the HP/LP comparisons likely reflect insufficient statistical power to discern significant correlations, as has been recently emphasized in studies of age-related CCL influences in human bone (Willett et al., 2022). Willett and co-workers (2022) noted that a key challenge in conducting these types of studies using human cadaveric tissues is the large inter-donor variability. They also noted that inter-individual heterogeneity may explain differences in findings between human studies that used 10–20 donors versus others with 50 or more. Hence, in those types of studies, larger sample sizes are needed to provide sufficient statistical power to detect strong correlations between the studied parameters. A similar issue might be at play in the present study; we report various statistical trends because they provide insights for future investigations.

**Fig. 5.**
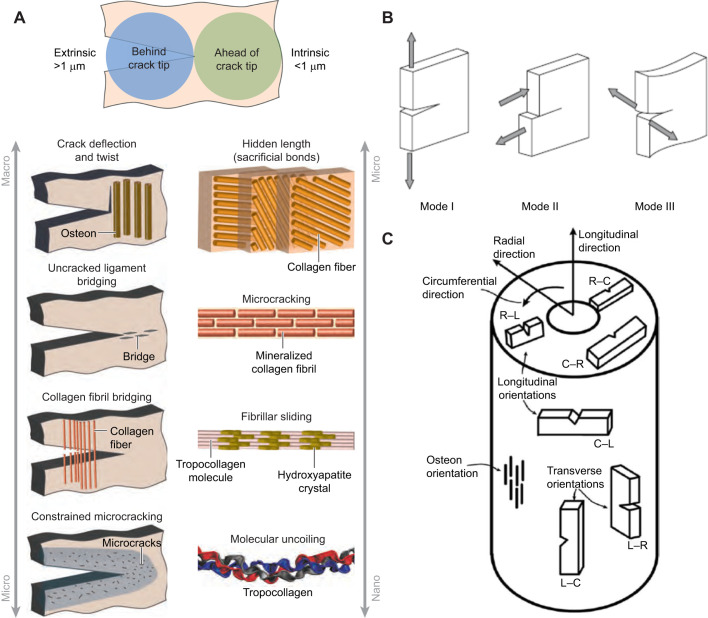
**Extrinsic and intrinsic toughening mechanisms, loading modes and ASTM E399 fracture toughness standard.** (A) The left side of this graphic primarily shows extrinsic toughening mechanisms in notched mechanical test specimens; these mechanisms include osteons and non-osteonal CFO. On the right are intrinsic toughening mechanisms, which include CCLs and, to some extent, also likely non-osteonal CFO. Figure reproduced from Launey et al. (2010) with permission of Annual Reviews, Inc. (B) Different modes of loading of notched specimens: mode I (opening mode), mode II (shear loading) and mode III (tearing and or anti-shear loading). (C) Orientations of notched specimens in accordance with ASTM E399 fracture toughness standard (1997, see Chittibabu et al., 2016). C, circumferential; L, longitudinal; R, radial. Figure reproduced from Chittibabu et al. (2016) with permission of Advances in Science and Technology Research Journal; Creative Commons Attribution 4.0 International (CC BY 4.0).

In natural/healthy conditions, bone is able to withstand complex physiological loading patterns by various fracture resistance mechanisms, both intrinsic and extrinsic (Launey et al., 2010). Intrinsic mechanisms include fibrillar sliding, dilatation bands, sacrificial bonds (includes CCLs), non-collagenous protein functions and molecular uncoiling (CCLs are likely involved here) (Siegmund et al., 2008; Depalle et al., 2015) (Fig. 5A, bottom right). Extrinsic mechanisms include microcrack deflection and twist at cement lines of osteons (osteon collagen/lamellar morphotypes are involved here), uncracked segment bridging and collagen fibril bridging (left in Fig. 5A). These mechanisms are deemed beneficial because they absorb energy (Launey et al., 2010). [Regarding the beneficial aspects of microcrack formation and propagation, Burr (2011) states: ‘Although the growth of microcracks in bone, which will increase the apparent microdamage burden, is widely viewed as a negative effect on bone's mechanical properties (and is), it actually delays or prevents the ultimate failure of the bone by releasing energy that otherwise would lead to immediate bone fracture. Easier crack initiation in this case is an adaptation to prevent the early failure of more heavily glycated [where PE CCLs are more prevalent], and less ductile, bone. Thus, crack accumulation, whether caused by pharmaceutical treatments that reduce remodeling, or caused by overuse during athletic and military exercises, is an adaptive mechanism to dissipate energy and delay fracture. This is especially true if crack growth can be constrained by the heterogeneous microstructure of the bone, i.e. by interfaces such as [osteonal] cement lines that will allow the crack to dissipate energy, but prevent it from growing to critical size.’] There is mutual competition between what can be termed intrinsic dam­age processes that operate ahead of the tip of a microcrack to promote its beneficial propagation, and extrinsic microcrack-tip-shielding mechanisms that act mostly behind the microcrack tip to inhibit deleterious expansion/propagation (Fig. 5A, top) (Ritchie, 2011). Our findings show that HP, LP and HP/LP (but not PE) influence the mechanical behavior of the equine MC3 in SMS compression tests. This is noteworthy because it suggests that enzymatic CCLs are valuable intrinsic toughening mechanisms in this context. In contrast, characteristics that are considered extrinsic toughening mechanisms in bone include osteons and, likely, CFO of the osteonal and non-osteonal (i.e. primary) bone. In turn, osteon MTS would also be predicted to be important in extrinsic toughening because of the interdependence between osteon MTS and OPD. In the intrinsic versus extrinsic dichotomy, osteonal pullout is an important extrinsic energy-absorbing mechanism. However, our data suggest that osteon MTS is not important in this context – but CFO clearly is. As discussed below, additional testing regimes using notch specimens are needed to more fully explore potential micro- and macro-mechanical roles played by regional variation in osteon MTSs and also their potential mechanically relevant interplay with CCLs.

We find it intriguing that while CFO (which includes consideration of osteon and non-osteon CFO) was prominently correlated with energy absorption parameters in both SMS compression and SMS tension tests, osteon MTS did not correlate significantly with any of the mechanical parameters in these two testing modes (Table 4). This was an unexpected outcome of our study. The significant correlation between energy absorption and predominant CFO – and not osteon MTS – in both testing modes likely reflects the fact that there are fewer osteons in ∼30–50% of the sampled regions. In other words, regionally predominant CFO also occurs in the primary bone, which was the majority of the tested sample in a significant number of cases. Hence, the equine bones tested in the present study notably differ from the typically more highly remodeled (secondary osteonal) bone of adult humans. Consequently, a significant proportion of the regional CFO variation in the equine MC3, including the more fracture prone dorsal–lateral cortex of racing animals, occurs within the primary (non-osteonal) bone. Thus, the primary bone also exhibits preferred CFO (i.e. longitudinal CFO in tension-adapted regions and oblique-to-transverse CFO in compression-adapted regions) as also shown in an ontogenetic study of sheep radii (Skedros and Kuo, 1999; Skedros, 2024). It is speculated that regional variation in osteon morphotypes will have more substantial mechanical relevance in bones that are more highly remodeled (i.e. relatively higher FASB and OPD than seen in our equine MC3 samples), including specific bones in humans that are prone to stress fractures such as the femur, tibia and metatarsals.

Our data pave the way for additional studies on extrinsic and intrinsic toughening mechanisms in the context of SMS testing. Future studies aimed at achieving a better understanding of the relationship between bone toughness, CCLs and osteon MTSs in equine MC3s must evaluate notched specimens that allow for controlled crack propagation in accordance with ASTM standards (Koester et al., 2008; Chittibabu et al., 2016; Gauthier et al., 2018) (Fig. 5B,C). SMS testing in tension and generally larger sample sizes are also needed in future studies that aim to gain a greater understanding of potential influences of CCLs and osteon MTSs in this loading mode when compared with SMS compression testing. More studies are needed that specifically evaluate the mechanical consequences of differences in the distributions of specific osteon morphotypes, similar to the work of Bigley et al. (2006) and Hiller et al. (2003).

Bigley et al. (2006) advanced the mechanical pushout methods pioneered by Ascenzi and Bonucci (1972) and reported important details of the mechanical properties of individual osteons in isolation and with consideration of the histomorphology of their surrounding matrix. Using osteons from the diaphysis of the MC3 of an adult Thoroughbred horse, Bigley et al. (2006) revealed clear differences in interfacial strength (at osteon cement line and interlamellar interfaces) associated with variations in CFO that typify the four osteon morphotypes that they evaluated. Overall, bright osteons (‘compression adapted’) had the highest interfacial debonding strength (40.3 MPa), and dark osteons (‘tension adapted’, Fig. 1) had the lowest strength (22.8 MPa) (*P*<0.05). In terms of maximum interfacial shear stress, the bright osteons also had highest stress (82.6 MPa) and the dark osteons had the least (63.6 MPa) (*P*<0.05).

The linear regression analysis of Bigley et al. (2006) also showed that the histomorphology of the surrounding matrix also profoundly influenced the shear strength of the osteons. The relationship of osteon MTS with shear (the ‘third strain mode’) is an important consideration in future studies because it has been hypothesized that hybrid osteon morphotypes (‘h’ in Fig. 1) preferentially accommodate shear stresses (Skedros et al., 2011a). However, a specific MTS scheme that adequately includes these putative shear-adapted ‘hybrids’ has not been developed. In summary, their study shows that osteon collagen/lamellar morphotypes play an important role in bone mechanics in ways that cannot be detected using the methods employed in the present study. In equine MC3s, Hiller et al. (2003) showed that osteon debonding and pullout are important toughening mechanisms, which likely help avoid progression to ultimate failure when the bone tissue experiences yielding in natural conditions. Loads that are near yield-stress levels (before yielding occurs) are known to cause microscopic damage and the energy absorbed by this damage helps keep the bone intact (Reilly, 2000).

As noted by Burr (2011), studies have shown that reduced bone remodeling (i.e. reduced formation of new osteons) allows the formation of additional collagen cross-links by non-enzymatic means as a consequence of bone matrix glycation and the accumulation in bone tissue of what are known as ‘advanced glycation end-products’ (AGEs) (Allen et al., 2008). The non-enzymatic CCL that we studied (PE) is an often-studied AGE (Willett et al., 2022). The accumulation of AGEs is directly related to the rate of bone turnover, and is the result of the failure to renew the tissue – hence their accumulation with aging. Bone glycation allows microcracks in bone to grow more easily, therefore increasing the apparent microdamage burden and has the added effect of reducing the post-yield deformation of bone (Vashishth et al., 2001; Vashishth, 2009), which makes the bone tissue more brittle and more likely to fracture (Tang et al., 2009; Burr, 2011). Although the FASB and OPD measurements made in the present study do not reflect the actual rate of remodeling, the positive/significant correlation of each of these characteristics with LP is an intriguing finding. Fluorochrome labeling studies (which allow for determination of osteonal remodeling rate and an estimation of mean tissue age) would be an important avenue to also consider in future studies that examine regional variation and the putative mechanical roles of CCLs and osteon MTSs in equine MC3s. Such studies would also help determine whether some of the significant and trend correlations between material characteristics found in the present study are related to mean tissue age. For example, they would also help determine whether the significant correlation of osteon MTS with % ash (*r*=0.47, *P*=0.02) (Table S3) might be related to the possibility that the oldest (more highly mineralized) osteons tend to be brighter (more oblique-to-transverse CFO) in polarized light. Larger sample sizes that include more horses with advanced age would likely be needed to detect age-related changes in CCLs, especially for detecting age-related mechanical consequences of the accumulation of PE in this species.

In a study dealing with enzymatic and non-enzymatic CCL densities from 32 paired human femoral diaphyses, femoral necks and radial diaphyses, Gauthier and co-workers (2018) found large significant differences between these three regions. However, the difference between the medial and lateral regions of the femoral diaphysis was relatively small, 8%, which they suggested demonstrated that CCL content is not dependent on the region of the femoral diaphysis. This conclusion in turn suggests that adaptations mediated by CCLs for SMS differences between the medial femoral region (typically considered a habitual ‘compression region’) and lateral femoral region (typically considered a habitual ‘tension region’) are not that important. But we have argued that the majority of the diaphyseal region of the human femur receives prevalent/predominant torsion (which engenders diffuse shear strains) that nullifies a habitual directionally consistent bending strain distribution that is seen in other more simply loaded bones (e.g. equine MC3s, and equine and sheep radii) or bone regions (e.g. subtrochanteric human femur) (Skedros, 2024). In other words, abundant, though indirect, evidence (adequate *in vivo* strain data are not available from human femora) supports the conclusion that SMS differences in terms of net tension versus net compression in mutually exclusive regions do not exist in the human femoral mid-diaphyseal region (Skedros, 2024). In contrast, as shown in Figs 2 and 3, the equine MC3 does experience habitual directionally consistent bending, though with some torsion (see neutral axis rotation in Fig. 2) that increases the complexity of loading more so than seen in the equine and sheep radius (Skedros, 2024). Notably, what we consider the ‘compression-region’ versus ‘tension-region’ difference in HP in the equine MC3 (shown as statistically significant and trend differences in Fig. 4B) is ∼23%, in contrast to the 8% reported by Gauthier and co-workers (2018) in the human femoral diaphysis in the presumed SMS context (i.e. net tension versus net compression in opposing cortices). Additionally, the percentage difference between the dorsal–medial and lateral regions in the present study was 25% for CFO and 17% for MTS (Fig. 4A). These differences seem to be SMS because the majority of our MC3s showed at least a 20% difference in these two characteristics in the dorsal–medial versus lateral comparison (Skedros et al., 2006). However, the absence of differences in CFO and MTS in the dorsal–medial versus dorsal–lateral comparison is unexpected – the majority of the bones had <9% difference in this specific comparison. This unexpected outcome might reflect heterogeneity in ambulatory activities of our animals and hence heterogeneity of the strain distributions of their MC3s, which would confound SMS adaptations in this model. This possibility seems consistent with the fact that Biewener and co-workers (1983) did not find distinct net tension- and net compression-loaded regions in the MC3s of the relatively small horses (*n*=3) that they studied at trot and canter speeds. Hence, they did not detect a neutral axis in their MC3s, which starkly contrasts with the strain data of Gross and co-workers (1992) that is depicted in our Fig. 3 and clearly shows a neutral axis (i.e. net tension on one side and net compression on the other side of this axis). It is important to note that Biewener and co-workers (1983) used only two strain gauges at mid-diaphysis (one dorsal, one palmar) of non-racing horses whereas Gross and co-workers (1992) used three strain gauges at equidistant locations of a racehorse (three gauges is the minimum number needed for developing a robust finite element model). Gross and co-workers (1992) also studied the strain distribution of the equine MC3s at galloping speeds and found a neutral axis such as that shown in our Fig. 3.

Mechanical testing methods that are different from those used in the present study are needed to better elucidate the relative roles that CCLs have in affecting bone quality. For example, future studies should consider tests that: (1) measure damage accumulation or fatigue behavior that include additional complexities of physiological loading (Gibson et al., 1995; Joo et al., 2007) and (2) explore the intrinsic and extrinsic mechanisms that mediate differences between initiation and propagation toughness by using notched specimens (Fig. 5B,C). Studies that examine SMS tension-tested specimens for CCLs are also needed. Additionally, there are a variety of other CCLs and other types of post-translational CCL modifications that should be evaluated in future studies (Vashishth, 2009; Willett et al., 2022).

### Conclusions

Compression-tested specimens from various locations in mid-diaphyseal regions of mature non-racing equine MC3s were evaluated for CCLs. Tension-tested specimens could not be similarly analyzed because of insufficient material remaining from these specimens. Results showed that among the studied CCLs, only the enzymatic CCLs correlated significantly with mechanical parameters in these compression tests: LP with energy absorption, HP/LP with elastic modulus (both *r*=0.4). HP/LP showed a trend with energy absorption (*r*=−0.3, *P*=0.08). Notably, HP/LP more strongly correlated with osteon density and mineralization than CFO or MTS. These data support the hypothesis that CCLs contribute to adapting regions in equine MC3s for differences in load histories typified by ambient tension or compression. In contrast to our CCL analyses in only compression-tested specimens, predominant CFO, osteon MTSs and other non-CCL material characteristics were examined in specimens exclusively tested in tension (dumbbells) and compression (cubes). These results showed that predominant CFO more strongly correlated with energy absorption than MTS in both testing modes. In general, CFO was found to be relatively prominent in affecting regional toughness in these equine MC3s in compression and tension tests. Osteon MTSs likely have a subsidiary role in these contexts because of their relatively lower prevalence (30–50%) in many of the tested specimens. Hence, the greater importance of CFO likely reflects the fact that it was quantified in entire microscopic images that include osteonal and non-osteonal (primary) bone. Analyses of CCLs and other material characteristics in tension-tested specimens are needed to more fully understand how CCLs influence the mechanical properties of bone in regions that are subject to prevalent/predominant tension, compression and shear.

## Supplementary Material

10.1242/jexbio.247758_sup1 Supplementary information
