# Supplementary material for: Roles of collagen cross-links and osteon collagen/lamellar morphotypes in equine third metacarpals in tension and compression tests
Source: J Exp Biol. 2024 Jul 24;227(14):jeb247758. doi: 10.1242/jeb.247758 (PMC11418171; doi:10.1242/jeb.247758)
Supplement: Supplementary information [file jexbio-227-247758-s1.pdf]

Table S1. All Specimens Correlation Matrix

	Ash	Porosity	FASB	OPD	OA	OLPD	CFO	MTS	Pre-yield Energy	Post-yield Energy	Total Energy	Elastic Modulus	Yield Stress	Ultimate Stress	Age
**Ash**	----														
**Porosity**	----	----													
**FASB**	----	----	----												
**OPD**	----	----	**r = 0.91<br>p < 0.01<br>n = 88**	----											
**OA**	----	----	**r = 0.21<br>p = 0.04<br>n = 88**	----	----										
**OLPD**	----	**r = 0.21<br>p = 0.04<br>n = 88**	**r = -0.25<br>p = 0.02<br>n = 88**	----	----	----									
**CFO**	----	**r = 0.19<br>p = 0.09<br>n = 81**	**r = 0.60<br>p < 0.01<br>n = 81**	**r = 0.57<br>p < 0.01<br>n = 81**	----	----	----								
**MTS**	**r = 0.27<br>p = 0.02<br>n = 73**	----	**r = 0.25<br>p = 0.03<br>n = 73**	**r = 0.27<br>p = 0.02<br>n = 73**	----	----	**r = 0.63<br>p < 0.01<br>n = 73**	----							
**Pre-yield Energy**	----	----	**r = -0.19<br>p = 0.09<br>n = 83**	----	----	**r = -0.21<br>p = 0.06<br>n = 83**	----	----	----						
**Post-yield Energy**	----	----	**r = 0.32<br>p < 0.01<br>n = 80**	**r = 0.21<br>p = 0.06<br>n = 80**	**r = 0.19<br>p = 0.09<br>n = 80**	----	**r = 0.44<br>p < 0.01<br>n = 74**	**r = 0.31<br>p = 0.01<br>n = 66**	**r = -0.40<br>p < 0.01<br>n = 90**	----					
**Total Energy**	----	----	**r = 0.27<br>p = 0.01<br>n = 81**	----	**r = 0.22<br>p = 0.05<br>n = 81**	----	**r = 0.44<br>p < 0.01<br>n = 74**	**r = 0.28<br>p = 0.02<br>n = 66**	----	**r = 0.95<br>p < 0.01<br>n = 90**	----				
**Elastic Modulus**	**r = 0.24<br>p = 0.02<br>n = 93**	**r = -0.19<br>p = 0.09<br>n = 83**	----	----	----	----	----	**r = 0.21<br>p = 0.08<br>n = 68**	**r = -0.42<br>p < 0.01<br>n = 93**	**r = 0.23<br>p = 0.03<br>n = 90**	----	----			
**Yield Stress**	**r = 0.18<br>p = 0.08<br>n = 93**	**r = -0.30<br>p = 0.01<br>n = 83**	----	----	----	**r = -0.28<br>p = 0.01<br>n = 83**	----	----	**r = -0.67<br>p < 0.01<br>n = 93**	**r = -0.30<br>p < 0.01<br>n = 90**	----	**r = 0.32<br>p = 0.01<br>n = 93**	----		
**Ultimate Stress**	**r = 0.31<br>p < 0.01<br>n = 93**	**r = -0.38<br>p < 0.01<br>n = 83**	----	----	----	**r = -0.27<br>p = 0.01<br>n = 83**	----	----	**r = 0.61<br>p < 0.01<br>n = 93**	----	----	**r = 0.20<br>p = 0.05<br>n = 93**	**r = 0.85<br>p < 0.01<br>n = 93**	----	
**Age**	**r = 0.25<br>p = 0.02<br>n = 98**	----	**r = 0.56<br>p < 0.01<br>n = 88**	**r = 0.51<br>p < 0.01<br>n = 88**	----	**r = -0.44<br>p < 0.01<br>n = 88**	**r = 0.34<br>p < 0.01<br>n = 81**	----	----	----	----	----	----	----	----

Bold: $0.06 \leq p \leq 0.09$

Bold and grey highlight: $p \leq 0.05$

Journal of Experimental Biology • Supplementary information

Table S2A. Mechanical vs. Material Correlation Tables

SMS Compression Correlation Matrix (Includes Lateral)

	Ash	Porosity	FASB	OPD	OA	OLPD	CFO	MTS	HP	LP	PE	HP/LP	Age
**Pre-yield Energy**	----	----	----	----	----	**$r = -0.27$<br>$p = 0.09$<br>$n = 40$**	**$r = 0.44$<br>$p = 0.01$<br>$n = 35$**	----	----	----	----	----	----
**Post-yield Energy**	----	----	----	----	----	----	----	----	----	----	----	----	----
**Total Energy**	----	----	**$r = 0.36$<br>$p = 0.03$<br>$n = 38$**	----	----	----	**$r = 0.60$<br>$p < 0.01$<br>$n = 33$**	**$r = 0.36$<br>$p = 0.05$<br>$n = 29$**	----	**$r = 0.32$<br>$p = 0.06$<br>$n = 36$**	----	----	----
**Elastic Modulus**	----	----	----	----	**$r = -0.30$<br>$p = 0.06$<br>$n = 40$**	----	----	----	----	----	----	----	----
**Yield Stress**	----	----	----	----	----	----	----	----	----	----	----	----	----
**Ultimate Stress**	**$r = 0.49$<br>$p < 0.01$<br>$n = 40$**	**$r = -0.27$<br>$p = 0.09$<br>$n = 40$**	----	**$r = 0.33$<br>$p = 0.04$<br>$n = 40$**	----	**$r = -0.32$<br>$p = 0.05$<br>$n = 40$**	----	----	----	----	----	----	----

Bold: $0.06 \leq p \leq 0.09$

Bold and grey highlight: $p \leq 0.05$

Table S2B. SMS Compression Correlation Matrix (No Lateral)

	Ash	Porosity	FASB	OPD	OA	OLPD	CFO	MTS	HP	LP	PE	HP/LP	Age
**Pre-yield Energy**	----	----	**r = 0.37<br>p = 0.05<br>n = 30**	**r = 0.35<br>p = 0.06<br>n = 30**	----	**r = -0.42<br>p = 0.02<br>n = 30**	**r = 0.41<br>p = 0.03<br>n = 27**	----	----	----	----	----	----
**Post-yield Energy**	----	----	----	----	----	----	----	----	----	----	----	**r = -0.34<br>p = 0.08<br>n = 28**	----
**Total Energy**	----	----	**r = 0.44<br>p = 0.01<br>n = 30**	----	----	----	**r = 0.57<br>p < 0.01<br>n = 27**	----	----	**r = 0.40<br>p = 0.03<br>n = 28**	----	**r = -0.33<br>p = 0.08<br>n = 28**	----
**Elastic Modulus**	**r = 0.45<br>p = 0.01<br>n = 30**	----	----	----	----	**r = -0.40<br>p = 0.03<br>n = 30**	----	----	----	----	----	**r = 0.40<br>p = 0.04<br>n = 28**	----
**Yield Stress**	**r = 0.40<br>p = 0.03<br>n = 30**	----	----	**r = 0.35<br>p = 0.06<br>n = 30**	----	**r = -0.50<br>p = 0.01<br>n = 30**	----	----	----	----	----	----	----
**Ultimate Stress**	**r = 0.51<br>p < 0.01<br>n = 30**	**r = -0.31<br>p = 0.09<br>n = 30**	**r = 0.33<br>p = 0.07<br>n = 30**	**r = 0.36<br>p = 0.05<br>n = 30**	----	**r = -0.49<br>p = 0.01<br>n = 30**	----	----	----	----	----	**r = 0.33<br>p = 0.09<br>n = 28**	----

Bold: $0.06 \le p \le 0.09$

Bold and grey highlight: $p \le 0.05$

Journal of Experimental Biology • Supplementary information

Table S2C. SMS Tension Correlation Matrix

	Ash	Porosity	FASB	OPD	OA	OLPD	CFO	MTS	Age
**Pre-yield Energy**	----	**r = -0.73 p = 0.02 n = 9**	----	----	**r = 0.61 p = 0.08 n = 9**	----	----	----	----
**Post-yield Energy**	----	**r = -0.62 p = 0.08 n = 9**	----	----	----	**r = -0.71 p = 0.03 n = 9**	**r = 0.73 p = 0.03 n = 9**	----	----
**Total Energy**	----	**r = -0.67 p = 0.05 n = 9**	----	----	----	**r = -0.71 p = 0.03 n = 9**	**r = 0.72 p = 0.03 n = 9**	----	----
**Elastic Modulus**	----	**r = -0.76 p = 0.02 n = 9**	----	----	**r = 0.68 p = 0.04 n = 9**	**r = -0.73 p = 0.03 n = 9**	----	----	----
**Yield Stress**	----	**r = -0.83 p = 0.01 n = 9**	----	----	**r = 0.71 p = 0.03 n = 9**	**r = -0.68 p = 0.04 n = 9**	----	----	----
**Ultimate Stress**	----	**r = -0.82 p = 0.01 n = 9**	----	----	**r = 0.63 p = 0.07 n = 9**	**r = -0.77 p = 0.02 n = 9**	----	----	----

Bold: 0.06 ≤ p ≤ 0.09

Bold and grey highlight: p ≤ 0.05

Journal of Experimental Biology • Supplementary information

Table S2D. Non-SMS Compression Correlation Matrix (Includes Lateral)

	Ash	Porosity	FASB	OPD	OA	OLPD	CFO	MTS	HP	LP	PE	HP/LP	Age
**Pre-yield Energy**	**r = 0.24<br>p = 0.06<br>n = 60**	----	----	----	----	----	**r = 0.30<br>p = 0.03<br>n = 53**	----	----	----	----	----	----
**Post-yield Energy**	**r = -0.30<br>p = 0.02<br>n = 57**	----	**r = 0.23<br>p = 0.08<br>n = 57**	----	----	----	**r = 0.39<br>p < 0.01<br>n = 51**	----	----	----	----	**r = -0.23<br>p = 0.09<br>n = 55**	----
**Total Energy**	**r = -0.24<br>p = 0.07<br>n = 58**	----	----	----	----	----	**r = 0.47<br>p < 0.01<br>n = 51**	**r = 0.31<br>p = 0.04<br>n = 45**	----	----	----	**r = -0.25<br>p = 0.06<br>n = 56**	----
**Elastic Modulus**	----	**r = -0.30<br>p = 0.02<br>n = 60**	----	----	**r = -0.30<br>p = 0.02<br>n = 60**	**r = -0.24<br>p = 0.06<br>n = 60**	**r = -0.26<br>p = 0.06<br>n = 53**	----	----	----	----	----	----
**Yield Stress**	**r = 0.24<br>p = 0.06<br>n = 60**	**r = -0.26<br>p = 0.04<br>n = 60**	----	----	----	----	----	----	----	----	----	----	----
**Ultimate Stress**	**r = 0.50<br>p < 0.01<br>n = 60**	**r = -0.33<br>p < 0.01<br>n = 60**	----	**r = 0.29<br>p = 0.03<br>n = 60**	----	----	----	----	----	----	----	----	**r = 0.24<br>p = 0.07<br>n = 60**

Bold: $0.06 \le p \le 0.09$

Bold and grey highlight: $p \le 0.05$

Journal of Experimental Biology • Supplementary information

Table S2E. Non-SMS Compression Correlation Matrix (No Lateral)

	Ash	Porosity	FASB	OPD	OA	OLPD	CFO	MTS	HP	LP	PE	HP/LP	Age
**Pre-yield Energy**	**r = 0.39<br>p < 0.01<br>n = 50**	----	----	----	----	----	----	----	----	----	----	----	----
**Post-yield Energy**	**r = -0.33<br>p = 0.02<br>n = 50**	----	----	----	----	----	**r = 0.40<br>p < 0.01<br>n = 45**	----	----	----	----	**r = -0.28<br>p = 0.05<br>n = 48**	----
**Total Energy**	----	----	**r = 0.24<br>p = 0.09<br>n = 50**	----	----	----	**r = 0.45<br>p < 0.01<br>n = 45**	**r = 0.28<br>p = 0.08<br>n = 40**	**r = -0.25<br>p = 0.09<br>n = 48**	----	----	**r = -0.29<br>p = 0.05<br>n = 48**	----
**Elastic Modulus**	**r = 0.25<br>p = 0.09<br>n = 50**	**r = -0.40<br>p < 0.01<br>n = 50**	----	----	----	**r = -0.39<br>p < 0.01<br>n = 50**	**r = -0.26<br>p = 0.09<br>n = 45**	----	----	----	----	**r = 0.25<br>p = 0.09<br>n = 48**	----
**Yield Stress**	**r = 0.46<br>p < 0.01<br>n = 50**	**r = -0.38<br>p < 0.01<br>n = 50**	----	**r = 0.26<br>p = 0.07<br>n = 50**	----	**r = -0.35<br>p = 0.01<br>n = 50**	----	----	----	----	----	----	----
**Ultimate Stress**	**r = 0.52<br>p < 0.01<br>n = 50**	**r = -0.38<br>p = 0.01<br>n = 50**	----	**r = 0.30<br>p = 0.03<br>n = 50**	----	**r = -0.30<br>p = 0.04<br>n = 50**	----	----	----	----	----	----	**r = 0.27<br>p = 0.06<br>n = 50**

Bold: $0.06 \leq p \leq 0.09$

Bold and grey highlight: $p \leq 0.05$

Journal of Experimental Biology • Supplementary information

Table S2F. Non-SMS Tension Correlation Matrix

	Ash	Porosity	FASB	OPD	OA	OLPD	CFO	MTS	Age
**Pre-yield Energy**	----	----	**r = 0.38 p = 0.07 n = 23**	----	**r = 0.44 p = 0.03 n = 23**	----	----	----	----
**Post-yield Energy**	----	**r = -0.37 p = 0.08 n = 23**	----	----	----	----	----	----	----
**Total Energy**	----	**r = -0.41 p = 0.05 n = 23**	----	----	----	----	----	----	----
**Elastic Modulus**	**r = 0.49 p < 0.01 n = 33**	**r = -0.36 p = 0.09 n = 23**	----	**r = -0.49 p = 0.02 n = 23**	**r = 0.40 p = 0.06 n = 23**	----	----	----	----
**Yield Stress**	----	**r = -0.42 p = 0.05 n = 23**	----	----	**r = 0.61 p < 0.01 n = 23**	----	----	----	----
**Ultimate Stress**	----	**r = -0.45 p = 0.03 n = 23**	----	----	**r = 0.58 p < 0.01 n = 23**	----	----	----	----

Bold: $0.06 \leq p \leq 0.09$

Bold and grey highlight: $p \leq 0.05$

Journal of Experimental Biology • Supplementary information

Table S3A. Material vs. Material Correlation Tables

SMS Compression Correlation Matrix (1 to 1; w/ Lateral)

	Ash	Porosity	FASB	OPD	OA	OLPD	CFO	MTS	HP	LP	PE	HP/LP	Age
**Ash**	----												
**Porosity**	----	----											
**FASB**	----	----	----										
**OPD**	----	----	**r = 0.91<br>p < 0.01<br>n = 40**	----									
**OA**	----	----	**r = 0.34<br>p = 0.03<br>n = 40**	----	----								
**OLPD**	----	----	**r = -0.49<br>p < 0.01<br>n = 40**	**r = -0.49<br>p < 0.01<br>n = 40**	----	----							
**CFO**	----	----	**r = 0.51<br>p < 0.01<br>n = 35**	**r = 0.48<br>p < 0.01<br>n = 35**	----	----	----						
**MTS**	**r = 0.34<br>p = 0.06<br>n = 31**	----	----	----	----	----	**r = 0.49<br>p > 0.01<br>n = 31**	----					
**HP**	----	----	----	----	----	----	----	----	----				
**LP**	----	----	----	----	----	----	----	----	**r = 0.45<br>p < 0.01<br>n = 38**	----			
**PE**	----	----	----	----	----	----	**r = 0.31<br>p = 0.08<br>n = 33**	----	----	----	----		
**HP/LP**	**r = 0.37<br>p = 0.02<br>n = 38**	----	**r = -0.40<br>p = 0.01<br>n = 38**	**r = -0.37<br>p = 0.02<br>n = 38**	----	----	----	----	**r = 0.54<br>p < 0.01<br>n = 38**	**r = -0.47<br>p < 0.01<br>n =38**	----	----	
**Age**	**r = 0.31<br>p = 0.05<br>n = 40**	----	**r = 0.59<br>p < 0.01<br>n = 40**	**r = 0.58<br>p < 0.01<br>n = 40**	----	**r = -0.43<br>p = 0.01<br>n = 40**	**r = 0.30<br>p = 0.08<br>n = 35**	----	----	----	----	----	----

Bold: 0.06 ≤ p ≤ 0.09

Bold and grey highlight: p ≤ 0.05

Journal of Experimental Biology • Supplementary information

Table S3B. SMS Compression Correlation Matrix (1 to 1; No Lateral)

	Ash	Porosity	FASB	OPD	OA	OLPD	CFO	MTS	HP	LP	PE	HP/LP	Age
**Ash**	----												
**Porosity**	----	----											
**FASB**	----	----	----										
**OPD**	----	----	**r = 0.90<br>p < 0.01<br>n = 30**	----									
**OA**	----	----	**r = 0.32<br>p = 0.08<br>n = 30**	----	----								
**OLPD**	----	----	**r = -0.46<br>p = 0.01<br>n = 30**	**r = -0.48<br>p < 0.01<br>n = 30**	----	----							
**CFO**	----	----	**r = 0.67<br>p < 0.01<br>n = 27**	**r = 0.61<br>p < 0.01<br>n = 27**	----	----	----						
**MTS**	**r = 0.47<br>p = 0.02<br>n = 24**	----	----	----	----	----	**r = 0.38<br>p = 0.07<br>n = 24**	----					
**HP**	----	----	----	----	----	----	**r = -0.35<br>p = 0.08<br>n = 25**	----	----				
**LP**	----	----	----	----	----	----	----	----	**r = 0.45<br>p = 0.02<br>n = 28**	----			
**PE**	----	----	----	----	----	----	**r = 0.36<br>p = 0.07<br>n = 25**	----	----	----	----		
**HP/LP**	**r = 0.37<br>p = 0.05<br>n = 28**	----	**r = -0.51<br>p < 0.01<br>n = 28**	**r = -0.44<br>p = 0.02<br>n = 28**	----	----	**r = -0.34<br>p = 0.09<br>n = 25**	----	**r = 0.48<br>p = 0.01<br>n = 28**	**r = -0.53<br>p < 0.01<br>n = 28**	----	----	
**Age**	----	----	**r = 0.53<br>p < 0.01<br>n = 30**	**r = 0.57<br>p < 0.01<br>n = 30**	----	**r = -0.38<br>p = 0.04<br>n = 30**	**r = 0.35<br>p = 0.08<br>n = 27**	----	----	----	----	----	----

Bold: $0.06 \leq p \leq 0.09$

Bold and grey highlight: $p \leq 0.05$

Journal of Experimental Biology • Supplementary information

Table S3C. SMS Tension Correlation Matrix (1 to 1)

	Ash	Porosity	FASB	OPD	OA	OLPD	CFO	MTS	Age
**Ash**	----								
**Porosity**	----	----							
**FASB**	----	----	----						
**OPD**	**r = -0.50<br>p = 0.07<br>n = 14**	----	**r = 0.86<br>p < 0.01<br>n = 14**	----					
**OA**	----	----	----	**r = -0.73<br>p < 0.01<br>n = 14**	----				
**OLPD**	----	----	----	----	----	----			
**CFO**	----	----	**r = 0.67<br>p < 0.01<br>n = 14**	**r = 0.61<br>p = 0.02<br>n = 14**	----	----	----		
**MTS**	----	----	----	**r = 0.47<br>p = 0.09<br>n = 14**	**r = -0.64<br>p = 0.01<br>n = 14**	----	----	----	
**Age**	----	----	**r = 0.52<br>p = 0.06<br>n = 14**	----	----	----	----	----	----

Bold: 0.06 ≤ p ≤ 0.09

Bold and grey highlight: p ≤ 0.05

Journal of Experimental Biology • Supplementary information

Table S3D. Non-SMS Compression Correlation Matrix (1 to 1; w/ Lateral)

	Ash	Porosity	FASB	OPD	OA	OLPD	CFO	MTS	HP	LP	PE	HP/LP	Age
**Ash**	----												
**Porosity**	----	----											
**FASB**	----	----	----										
**OPD**	----	----	**r = 0.91<br>p < 0.01<br>n = 60**	----									
**OA**	----	----	**r = 0.27<br>p = 0.04<br>n = 60**	----	----								
**OLPD**	----	----	**r = -0.40<br>p < 0.01<br>n = 60**	**r = -0.28<br>p = 0.03<br>n = 60**	----	----							
**CFO**	**r = -0.23<br>p = 0.09<br>n = 53**	**r = 0.30<br>p = 0.03<br>n = 53**	**r = 0.56<br>p < 0.01<br>n = 53**	**r = 0.50<br>p < 0.01<br>n = 53**	**r = 0.27<br>p = 0.05<br>n = 53**	----	----						
**MTS**	----	----	----	----	----	----	**r = 0.57<br>p < 0.01<br>n = 47**	----					
**HP**	----	----	----	----	----	----	----	----	----				
**LP**	**r = -0.25<br>p = 0.06<br>n = 58**	----	**r = 0.36<br>p < 0.01<br>n = 58**	**r = 0.39<br>p < 0.01<br>n = 58**	----	----	**r = 0.27<br>p = 0.06<br>n = 51**	----	**r = 0.49<br>p < 0.01<br>n = 58**	----			
**PE**	----	----	----	----	----	----	**r = 0.24<br>p = 0.09<br>n = 51**	----	----	----	----		
**HP/LP**	**r = 0.28<br>p = 0.03<br>n = 58**	----	**r = -0.46<br>p < 0.01<br>n = 58**	**r = -0.46<br>p < 0.01<br>n = 58**	----	----	**r = -0.29<br>p = 0.04<br>n = 51**	----	**r = 0.51<br>p < 0.01<br>n = 58**	**r = -0.47<br>p < 0.01<br>n = 58**	----	----	
**Age**	----	----	**r = 0.63<br>p < 0.01<br>n = 60**	**r = 0.58<br>p < 0.01<br>n = 60**	----	**r = -0.44<br>p < 0.01<br>n = 60**	**r = 0.29<br>p = 0.03<br>n = 53**	----	----	----	----	----	----

Bold: 0.06 ≤ p ≤ 0.09

Bold and grey highlight: p ≤ 0.05

Journal of Experimental Biology • Supplementary information

Table S3E. Non-SMS Compression Correlation Matrix (1 to 1; No Lateral)

	Ash	Porosity	FASB	OPD	OA	OLPD	CFO	MTS	HP	LP	PE	HP/LP	Age
**Ash**	----												
**Porosity**	----	----											
**FASB**	----	----	----										
**OPD**	----	----	**r = 0.91<br>p < 0.01<br>n = 50**	----									
**OA**	----	----	**r = 0.25<br>p = 0.08<br>n = 50**	----	----								
**OLPD**	----	----	**r = -0.36<br>p = 0.01<br>n = 50**	----	----	----							
**CFO**	----	**r = 0.30<br>p = 0.05<br>n = 45**	**r = 0.67<br>p < 0.01<br>n = 45**	**r = 0.60<br>p < 0.01<br>n = 45**	**r = 0.31<br>p = 0.04<br>n = 45**	----	----						
**MTS**	**----**	----	----	----	----	----	**r = 0.48<br>p < 0.01<br>n = 40**	----					
**HP**	----	----	----	----	----	----	----	----	----				
**LP**	**r = -0.27<br>p = 0.07<br>n = 48**	----	**r = 0.39<br>p < 0.01<br>n = 48**	**r = 0.39<br>p < 0.01<br>n = 48**	----	----	----	----	**r = 0.50<br>p < 0.01<br>n= 48**	----			
**PE**	----	----	----	----	----	----	**r = 0.28<br>p = 0.07<br>n = 43**	----	----	----	----		
**HP/LP**	**r = 0.26<br>p = 0.07<br>n = 48**	----	**r = -0.54<br>p < 0.01<br>n = 48**	**r = -0.51<br>p < 0.01<br>n = 48**	----	----	**r = -0.39<br>p < 0.01<br>n = 43**	----	**r – 0.47<br>p < 0.01<br>n = 48**	**r = -0.49<br>p < 0.01<br>n = 48**	----	----	
**Age**	----	----	**r = 0.61<br>p < 0.01<br>n = 50**	**r = 0.58<br>p < 0.01<br>n = 50**	----	**r = -0.41<br>p < 0.01<br>n = 50**	**r = 0.32<br>p = 0.03<br>n = 45**	----	----	----	----	----	----

Bold: $0.06 \le p \le 0.09$

Bold and grey highlight: $p \le 0.05$

Journal of Experimental Biology • Supplementary information

Table S3F. Non-SMS Tension Correlation Matrix (1 to 1)

	Ash	Porosity	FASB	OPD	OA	OLPD	CFO	MTS	Age
**Ash**	----								
**Porosity**	----	----							
**FASB**	**r = -0.36<br>p = 0.06<br>n = 28**	----	----						
**OPD**	**r = -0.32<br>p = 0.10<br>n = 28**	----	**r = 0.84<br>p < 0.01<br>n = 28**	----					
**OA**	----	----	----	**r = -0.59<br>p < 0.01<br>n = 28**	----				
**OLPD**	----	----	**r = -0.34<br>p = 0.07<br>n = 28**	----	----	----			
**CFO**	----	----	**r = 0.47<br>p = 0.01<br>n = 28**	**r = 0.45<br>p = 0.02<br>n = 28**	----	----	----		
**MTS**	**r = 0.55<br>p < 0.01<br>n = 26**	----	----	----	**r = -0.35<br>p = 0.08<br>n = 26**	----	**r = 0.59<br>p < 0.01<br>n = 26**	----	
**Age**	**r = 0.31<br>p = 0.06<br>n = 38**	----	**r = 0.51<br>p < 0.01<br>n = 28**	**r = 0.40<br>p = 0.03<br>n = 28**	----	**r = -0.54<br>p < 0.01<br>n = 28**	**r = 0.37<br>p = 0.05<br>n = 28**	----	----

Bold: 0.06 ≤ p ≤ 0.09

Bold and grey highlight: p ≤ 0.05

Journal of Experimental Biology • Supplementary information

Table S4A. Strongest Significant Correlates in SMS Testing ‡

(mechanical parameters *vs*. material characteristics)

SMS Compression (includes palmar-medial and dorsal-medial cortices)

Mechanical property	N	Strongest	N	Second strongest
**Pre-yield Energy**	30	OLPD<br>r = -0.42	27	CFO<br>r = 0.41
**Post-yield Energy**	28	HP/LP<br>r = -0.34<br>p = 0.08*	----	----
**Total Energy**	27	CFO<br>r = 0.56	30	FASB<br>r = 0.44
**Elastic Modulus**	30	Ash<br>r = 0.45	28	HP/LP<br>r = 0.40
**Yield Stress**	30	OLPD<br>r = -0.50	30	Ash<br>r = 0.40
**Ultimate Stress**	30	Ash<br>r = 0.51	30	OLPD<br>r = -0.49

SMS Tension (only includes dorsal-lateral cortex)

Mechanical property	N	Strongest	N	Second strongest
**Pre-yield Energy**	9	Porosity<br>r = -0.74	9	OA<br>r = 0.61
**Post-yield Energy**	9	CFO<br>r = 0.73	9	OLPD<br>r = -0.71
**Total Energy**	9	CFO<br>r = 0.72	9	OLPD<br>r = -0.71
**Elastic Modulus**	9	Porosity<br>r = -0.76	9	OLPD<br>r = -0.73
**Yield Stress**	9	Porosity<br>r = -0.83	9	OLPD<br>r = -0.68
**Ultimate Stress**	9	Porosity<br>r = -0.82	9	OLPD<br>r = -0.80

***** Indicates statistical trends in three cases with exact p values shown. All other comparisons shown are ≤ 0.05. Blank cells indicate that no other correlations were at p ≤ 0.1.

Journal of Experimental Biology • Supplementary information

Table S4B. Strongest Significant Correlates in SMS Testing ‡
(material *vs*. material characteristics)

SMS Compression **(includes palmar-medial and dorsal-medial cortices)**

Material Property	N	Strongest	N	Second strongest	N	Third strongest
**Ash**	24	MTS<br>r = 0.47	28	HP/LP<br>r = 0.37	----	----
**Porosity**	----	----	----	----	----	----
**FASB**	30	OPD<br>r = 0.90	27	CFO<br>r = 0.67	28	HP/LP<br>r = -0.51
**OPD**	30	FASB<br>r = 0.90	27	CFO<br>r = 0.61	30	OLPD<br>r = -0.48
**OA**	30	FASB<br>r = 0.32<br>p = 0.08*	----	----	----	----
**OLPD**	30	OPD<br>r = -0.48	30	FASB<br>r = -0.46	27	CFO<br>r = -0.33<br>p = 0.10*
**CFO**	27	FASB<br>r = 0.67	27	OPD<br>r = 0.61	24	MTS<br>r = 0.38<br>p = 0.07*
**MTS**	24	Ash<br>r = 0.47	24	CFO<br>r = 0.38<br>p = 0.07*	22	LP<br>r = -0.36<br>p = 0.10*
**HP**	28	HP/LP<br>r = 0.48	28	LP<br>r = 0.45	25	CFO<br>r = -0.35<br>p = 0.08*
**LP**	28	HP/LP<br>r = -0.53	28	HP<br>r = 0.45	22	MTS<br>r = -0.36<br>p = 0.10*
**PE**	25	PE<br>r = 0.37<br>p = 0.07*	----	----	----	----

Journal of Experimental Biology • Supplementary information

**HP/LP**	28	LP<br>r = -0.53	28	FASB<br>r = -0.51	28	HP<br>r = 0.48

SMS Tension (only includes dorsal-lateral cortex)

Material Property	N	Strongest	N	Second strongest
**Ash**	14	OPD<br>r = -0.50<br>p = 0.07*	----	----
**Porosity**	----	----	----	----
**FASB**	14	OPD<br>r = 0.86	14	CFO<br>r = 0.67
**OPD**	14	FASB<br>r = 0.86	14	OA<br>r = -0.73
**OA**	14	OPD<br>r = -0.73	14	MTS<br>r = -0.64
**OLPD**	----	----	----	----
**CFO**	14	FASB<br>r = 0.67	14	OPD<br>r = 0.61
**MTS**	14	OA<br>r = -0.64	14	OPD<br>r = 0.46<br>p = 0.09*

***** Indicates statistical trends in three cases with exact p values shown. All other comparisons shown are ≤ 0.05. Blank cells indicate that no other correlations were at $p \leq 0.1$.

Journal of Experimental Biology • Supplementary information
